# Serum and Ascitic Fluid Superoxide Dismutase and Malondialdehyde Levels in Patients with Cirrhosis

**DOI:** 10.4137/bmi.s639

**Published:** 2008-03-12

**Authors:** Seren Ozenirler, Banu Sancak, Ugur Coskun

**Affiliations:** 1 Gastroenterology, Gazi University Medical School, Ankara, Turkey; 2 Biochemistry, Gazi University Medical School, Ankara, Turkey; 3 Internal Medicine, Gazi University Medical School, Ankara, Turkey

**Keywords:** ascitic fluid, liver cirrhosis, malondialdehyde, superoxide dismutase

## Abstract

**Summary:**

Serum and ascitic fluid superoxide dismutase (SOD) and malondialdehyde (MDA) levels were measured in 43 patients with cirrhosis and in a 10 healthy control group.

Compensated cirrhotic patients had no clinically detectable ascites, but decompensated patients had massive ascites. Cirrhotic patients were divided into three groups: patients with compensated cirrhosis (n = 16), patients with decompensated cirrhosis with Spontaneous bacterial peritonitis (SBP) (n = 14), and patients with decompensated cirrhosis without SBP (n = 13).

All cirrhotic patients in the experimental group had significantly higher serum SOD (p < 0.001) and MDA levels (p < 0.01) than those in the control group. There were no significant differences with respect to serum SOD and MDA levels among the three different groups of patients. There was no remarkable difference in ascitic fluid SOD and MDA levels between decompensated cirrhotic patients with and without SBP (p > 0.05).

These results suggest that the increase in serum SOD and MDA levels are not related to the presence of SBP and the status of liver cirrhosis. To sum up, clarifying the impact of increased serum SOD and MDA levels in cirrhotic patients needs further investigation.

## Introduction

It has been reported that the superoxide anion radical and its scavenger, SOD, play an important role in many diseases, such as: anemia, malignant lymphoma and infectious diseases (Abou-Seif Mam et al. 2000; Dias-Da-Motta et al. 1996; Liu et al. 1993, Pasguier et al. 1984). Increased production of free radicals plays an important role in the pathogenesis of liver disease and fibrosis (Ljubuncic et al. 2000; Nalini et al. 1999; Özenirler et al. 1994; Togashi et al. 1999). On the other hand, a lipid peroxidation product, MDA, affects lipocytes (Ito cells) directly or indirectly through the promotion of fibrogenesis in the liver (Friedman, 1999; Svegliati-Baroni et al. 1999; Sukamato et al. 1993). Many clinical studies, revealed the fact that MDA, which is a lipid peroxidation product, increases in various liver diseases (Paradis et al. 1997). These findings point out the importance of MDA in the progession of Liver cirrhosis (LC). Moreover, little is known about the importance of SOD in LC, and the studies dealing with these results are controversial. According to our knowledge, there is no published report regarding the importance of ascitic fluid SOD and MDA with and without SBP in patients with LC. The aim of the this study is to evaluate serum and ascitic fluid SOD and MDA levels, and to determine whether the presence of SBP and the status of LC have any effect on serum and ascitic fluid SOD and MDA levels in cirrhotic patients.

## Materials and Methods

Forty-three patients (32 men and 11 women, mean age: 48 ± 3 years) with cirrhosis and a 10 healthy control group (5 men and 5 women, mean age: 47.4 ± 7.7 years) were included in this study. The study was conducted between 1999 and 2000. Liver cirrhosis was proven histologically in 14 cases and diagnosed on the basis of clinical, biochemical, imaging methods (ultrasound, CT) and endoscopic signs in other patients because of bleeding risk. Cirrhosis was related to post-hepatitis B in 16 patients, chronic alcohol intake in 14 patients, post-hepatitis C in 8 patients and cryptogenic cirrhosis in 5 patients. The severity of the disease was evaluated according to the Child-Pugh (Pugh et al. 1973) classification. Patients with congestive heart failure, renal failure (plasmaurea>15 mmol/landcreatinine>150 μmol/l), lung disease, malignancy, hepatic encephalopathy or gastrointestinal bleeding were excluded. Diuretic and antibiotic therapy were stopped during the two weeks before the study. Serum total bilirubin, alanine amino transferase (ALT), albumin, gamma glutamyl transferase (GGT) and prothrombine time (PT) were measured in all of the patients. Characteristics of patients and control subjects are shown in Table 1.

Serum and ascitic fluid SOD and MDA levels were measured in 43 patients with cirrhosis and a 10 healthy control group. Serum and ascitic fluid samples were obtained from the patients and serum samples were obtained from the control subjects after overnight fasting. Compensated cirrhotic patients had no clinically detectable ascites but decompensated patients had massive ascites. Patients were divided into three groups: patients with compensated cirrhosis (n = 16), patients with decompensated cirrhosis and SBP, as defined by a polymorphonuclear cell count in ascitic fluid of >250,000/ml and/or a positive culture associated with a clinical sign of SBP (n = 14), and patients with decompensated cirrhosis without SBP (n = 13). Cultures and polymorphonuclear cell counts were performed on the ascitic fluid. Seven out of 14 cases of SBP had positive cultures (three Escherichia coli, two Bacteroides fragilis, one Enterococcus faecalis and one Staphyolococcus aureus). The remaining cases were defined by a polymorphonuclear cell count in ascitic fluid of >250,000/ml.

SOD measurement: The whole blood and ascitic fluid were centrifuged and blood serum was carefully separated. We treated the samples with chloroform and ethanol. and after centrifugation, 0.5 ml supernatant was used for the assay. This assay for superoxide dismutase activitiy involves xanthine oxidase which was used as superoxide generator. One unit SOD is defined as the amount of the protein that inhibitis the rate of NBT reduction by 50% (IU/ml) (Sun et al. 1988).

Malondialdehyde measurement: The levels of malondialdehyde were determined in serum and ascitic fluid of patients using the thiobarbituric acid method. The results were obtained in mol/ml. (Yoshioka et al. 1979).

The results are presented as mean ±SD. The differences in results between patients and control subjects were compared using the Krushal-Wallis test. The Mann-Whitney ‘U’ test was used for intragroup comparisons. The Pearson test was used for correlation analysis. The significance was taken at p < 0.05.

## Results

There were no significant differences between the three groups of cirrhotic patients and control subjects in terms of age, sex and pathogenesis of cirrhosis (Table 1). All cirrhotic patients showed significant increases in serum SOD (p < 0.001) and MDA (p < 0.05) levels in comparison with those of control subjects. No significant difference was found among the different groups of patients in terms of serum SOD and MDA levels (Table 2). Ascitic fluid SOD and MDA levels were not different between decompensated cirrhotic patients with and without SBP (Table 3). Serum ALT levels were not different among three groups of patients. Serum total bilirubin levels were found to be lower in patients with compensated cirrhosis than those in patients with decompensated cirrhosis without SBP. Serum albumin levels were found to be higher in patients with compensated cirrhosis than in other patients. Serum PT measurements were found to be lower in patients with compensated cirrhosis than in other patients (p < 0.001). There was no diffference in serum ALT, albumin, total bilirubin levels and PT measurements of decompensated patients in terms of the SBP. Serum GGT levels were found to be lower in patients with decompensated cirrhosis and SBP than in other patients (Table 4).

## Discussion

In our study, we measured serum and ascitic fluid SOD and MDA levels in patients with LC and serum SOD and MDA levels in control subjects to investigate the effect of severity of liver damage and SBP on these levels.

The results of several reports regarding the evaluation of serum SOD in patients with various forms of liver diseases are controversial (Makarenko et al. 1989; Zhurkin et al. 1989; Yasuyama et al. 1988; Irshad et al. 1988).

Increased SOD levels were found by Makarenko et al. (1989) in persistent and active hepatitis or cirrhotic patients. They explained the increase in SOD as an adaptive reaction directed to the prevention of lipid peroxidation intensification and its injurious effect on the cellular structures.

Similar results for patients with acute and chronic viral hepatitis were shown by Zhurkin et al. (1989), who explained SOD activation in red blood cells by enzyme induction with the superoxide anion radical.

Yasuyama et al. (1988) found significantly higher plasma SOD activity in chronic hepatitis, autoimmune hepatitis, primary biliary cirrhosis and hepatocellular carcinoma groups than in the healthy control group. They explained the increased activity of SOD by increased release of injured hepatocytes.

In our study, we found results similar to Yasuyama et al. All cirrhotic patients had significanthy higher serum SOD and MDA levels than the control group. On the other hand, in serum and ascites, there are many kinds of substances, such as albumin, vitamin C and E, bilirubin or uric acid as well as SOD itself, that can scavenge superoxide radicals. In our study, we could not eliminate the effects of these SOD mimetic substances on our results by using our method (Inci et al. 2003; Kato et al. 2003). However, we think that SOD mimetic factors may have same effect on both control subjects and patients.

Hadi et al. (1999) found lower activities of SOD in the erythrocytes obtained from patients with cirrhosis compared with those obtained from control subjects. They concluded that although the enzymatic antioxidant defense system is significantly reduced in erythrocytes obtained from cirrhotic patients, this did not lead to further peroxidative reactions in the erythrocytes, possibly due to peroxidation of some cellular structures sensitive to peroxidative attack.

There was, however, an important indication of accelerated peroxidative reaction in the plasma of the cirrhotic patients, which possibly resulted from extracellular oxidant stress in these patients.

Chen et al. (1997) found that the blood levels of superoxide in decompensated cirrhotic patients were greater than in healthy controls or compensated cirrhotic patients. They concluded that the increase in blood levels of superoxide in decompensated cirrhotic patients is related to the impairment of liver function.

Our results were not compatible with these studies. Our study scored the degree of liver severity according to the classification of Child-Pugh. As seen in Table 1, most of patients had Child A disease in compensated patients, while patients with decompensated cirrhosis had predominantly Child C disease. We found no difference in serum levels of SOD and MDA among these three groups of patients. Moreover, we could not find any correlation between these levels and liver function tests including serum levels of GGT, ALT, total bilirubin, albumin and PT measurement among the groups. Our results did not confirm the relationship or causality between serum SOD and MDA levels and the status of liver cirrhosis by using Child-Pugh Score system.

We also investigated whether the presence of SBP has any effect on serum SOD and MDA levels. However, serum SOD and MDA levels were found not to be significantly different in patients with and without SBP.

Our study showed that it is not only the serum levels but also the ascitic fluid SOD and MDA levels that turned out not to be different in decompensated cirrhotic patients with or without SBP. It was not compatible with the results of other studies regarding peritonitis and inflammation (Goode and Webster, 1993; Konuko lu et al. 1999).

In conclusion, we found significantly higher serum SOD and MDA levels in all cirrhotic patients than in the controls. On the other hand, high levels of serum SOD and MDA do not seem to be associated with the presence of SBP and compensation of liver cirrhosis. Moreover, we could not find any correlation between these levels and liver function tests. Further studies are needed to explain the mechanism of this increase in serum SOD and MDA levels in cirrhosis.

## Figures and Tables

**Table 1:** Characteristics of the patients.

Patients	n	Sex (M/F)	Age (year)	Ascites	SBP*	** CC (A/B/C)	Etiology HB/CA/HC/CR***
Controls	10	6/4	47 ± 7	No	−	−	−
Patients with compensated cirrhosis	16	13/3	45 ± 13	No	−	14/2/0	6/5/2/3
Patients with decompensated cirrhosis with SBP	14	10/4	46 ± 9	Severe	+	0/1/13	5/5/3/1
Patients with decompensated cirrhosis without SBP	13	9/4	45 ± 10	Severe	−	0/2/11	5/4/3/1

* SBP: Spontaneous bacterial peritonitis,

** CC: child-pugh classification,

*** HB: Hepatitis B; CA: chronic alcholism; HC: hepatitis C; CR: cryptogenic.

**Table 2:** Serum SOD and MDA levels of healthy controls and cirrhotic patients.

Patients	n	Serum SOD levels (IU/ml) (mean ± SD)	Serum MDA levels (nmol/ml) (mean ± SD)
Controls	10	0.395 ± 0.41*	7.95 ± 2.19**
Patients with compensated cirrhosis	16	4.38 ± 2.03	15.1 ± 6.2
Patients with decompensated cirrhosis with SBP	14	3.71 ± 1.08	14.25 ± 6.3
Patients with decompensated cirrhosis without SBP	13	4.69 ± 1.95	16.41 ± 8.94

* p < 0.001,

** p < 0.05.

**Table 3:** Ascitic fluid SOD and MDA levels of cirrhotic patients with and without SBP.

Patients	n	Ascites SOD levels (IU/ml) (mean ± SD)	Ascites MDA levels (nmol/ml) (mean ± SD)
Patients with decompensated cirrhosis with SBP	14	2.21 ± 1.34	9.96 ± 4.43
Patients with decompensated cirrhosis without SBP	13	2.56 ± 1.00	7.65 ± 2.81

**Table 4:** Serum ALT, T. Bilirubin, albumin, PT and GGT levels of patients.

Patients	ALT (mean ± SD)	T. Bilirubin (mean ± SD)	Albumin (mean ± SD)	P T (mean ± SD)	GGT (mean ± SD)
Patients with compensated cirrhosis	45.63 ± 21.77	1.90 ± 1.10**	3.61 ± 0.5*	14.68 ± 1.2*	49.63 ± 16.42
Patients with decompensated cirrhosis with SBP	65.71 ± 47.44	2.82 ± 1.44	2.68 ± 0.54	18.32 ± 2.53	80.08 ± 48.99**
Patients with decompensated cirrhosis without SBP	67.31 ± 49.92	3.80 ± 3.13	2.77 ± 0.5	18.15 ± 3.7	62.9 ± 36.3

* p < 0.001,

** p < 0.05.
